# Malaria complicated by gangrene: a case presentation and review

**Published:** 2011-11-25

**Authors:** Faraj Omar Alkizim, Duncan Matheka, Otieno Walter Mwanda

**Affiliations:** 1 School of Medicine, University of Nairobi, PO Box 30197-00100, Nairobi, Kenya; 2 Department of Human Pathology, University of Nairobi, PO Box 30197-00100, Nairobi, Kenya

**Keywords:** Gangrene, malaria, complication, Kenya

## Abstract

Symmetrical peripheral gangrene (SPG) is an extremely rare complication of malaria. It occurs acutely and progresses rapidly to cause irreversible necrosis of tissue following which debridement or amputation is inevitable. We present a case of malaria complicated by SPG. A 54-year old male developed SPG two days after he was diagnosed with severe malaria and treated with intravenous quinine. Despite intervention quad-amputation was necessary as the gangrene had involved all four limbs. SPG secondary to malaria is caused by obstruction of arterioles following sequestration of parasite infected erythrocytes. This is extremely rare, hence almost never anticipated during management of malaria patients. Furthermore due to its rapid progression, it is almost always detected at an advanced irreversible stage. Physicians managing malaria should therefore be vigilant, and look out for SPG, as its prognosis is dependent on correct and timely intervention.

## Introduction

Symmetrical peripheral gangrene (SPG) was first described in 1891 [1]. It is now a well-documented, yet rare, disorder characterized by distal ischemic gangrene of two or more sites in the absence of large vessel obstruction or vasculitis [2]. Its pathogenesis is not well understood, and it has been linked to a wide variety of infective and non-infective etiological factors [3].

Malaria is one of the leading causes of morbidity and mortality worldwide. It is reported that 300 – 500 million people are infected by the parasite annually, leading to a death toll of 1.1-2.7 million [4]. It has been reported as one of causes of SPG [3], although extremely rare. Despite the millions of malaria cases annually, there has only been 23 cases, over the years, to the best of our knowledge, reported to have complicated with SPG (Table 1). We hereby describe the first (1^st^) such case in Kenya, third (3^rd^) in Africa, and twenty fourth (24^th^) worldwide.

**Table 1:** A review of all the 23 past similar cases of gangrene complicated severe malaria

Case	Reference	Age and gender	Country	Estimated Parasitemia (%)	Hb (g/dL)	Platelet count (x10^3^/ µl)	Clinical features	Antimalarial therapy	Anti-clotting therapy	Resolution
**1**	[25]	21, M	India	NA	6.8	210	DIC, dry gangrene of fingers and toes within 3 days	Artesunate	None	Amputated
**2**	[25]	59, M	India	NA	5.5	100	DIC, dry gangrene of fingers and toes within 3 days	Quinine	None	Resolved
**3**	[25]	35, F	India	NA	7.1	110	DIC, dry gangrene of both feet	Quinine	None	Resolved
**4**	[26]	11, M	Zimbabwe	NA	11.6	21	DIC, necrotic area on right hand	Chloroquine, Quinine	Heparin	Resolved
**5**	[26]	9, M	Zimbabwe	NA	12.9	10	Discoloration of digits on both feet	Quinine, Dexamethasone	Heparin, Streptokinase	Resolved
**6**	[27]	46, F	India	6	5	160	DIC, dry gangrene of both feet	Quinine	None	Refused amputation
**7**	[28]	13, F	Thailand	NA	7.6	46	Cerebral malaria, dry gangrene of toes within 3 days	Quinine	NA	Debridement
**8**	[28]	10, F	Thailand	NA	NA	<50	cerebral malaria, DIC, dry gangrene of toes within 3 days	Quinine	NA	Resolved
**9**	[29]	26, F	India	NA	10.0	NA	Dry gangrene of fingers and toes	Quinine	NA	Amputated
**10**	[30]	22, M	India	NA	13	190	Dry gangrene of fingers and toes	Quinine	NA	
**11**	[31]	56, F	European tourist to Sao Tome	10.3	13.9	13	cerebral malaria, DIC, dry gangrene of fingers and toes within 3 days	Quinine, Doxycycline	Heparin	Amputated
**12**	[32]	65, F	India	14	3.2	156	Cerebral malaria, DIC, Dry gangrene of left thigh	Quinine	NA	Amputated
**13**	[33]	2.5, F	India	90	6	80	Dry gangrene of fingers, toes, earlobes, and patches on arms and legs.	Quinine	NA	Debridement, resolved
**14**	[34]	10, F	India	>5	4.4	92	Dry gangrene of toes	Quinine	Heparin, Warfarin	Amputated
**15**	[35]	0.75, F	India	5	4	70	Dry gangrene of thigh, lower left abdominal wall, and gluteal region.	Quinine	NA	Debridement
**16**	[36]	40, M	Thailand	21	9.7	395	Dry gangrenous patches on lower limb	Artesunate	NA	Resolved
**17**	[36]	45, M	Thailand	20	12.3	23	Dry gangrene of toes	Artesunate, Mefloquine	NA	Amputated
**18**	[36]	59, F	Thailand	11	10	34	Dry gangrene of toes	Artesunate, Mefloquine	NA	Resolved
**19**	[37]	26, F	India	NA	9.75	230	Dry gangrene of toes within 8 days	Artesunate, cephalosporin	NA	Not stated
**20**	[38]	61, M	Austrian traveler to Uganda	NA	NA	5-15	DIC, toe dry gangrene within 3 days	Quinine, Mefloquine	NA	Amputation
**21**	[6]	44, F	UK traveler to Nigeria	24	11.8	48	DIC, dry digital gangrene on hands and feet within 9 days	Artesunate, Doxycycline	Heparin	Debridement
**22**	[39]	6, F	India	>90	5.6	84	Dry gangrene on toes extending to middle foot.	Artesunate, Mefloquine, Primaquine	Heparin, Warfarin	Amputated
**23**	[40]	12, M	India	21	7.0	166	Dry gangrene of lower limbs extending to the mid-calf.	Quinine	NA	Refused amputation
**24**	The current case	54, M	Ugandan referred to Kenya	NA	9.03	>450	Dry digital gangrene on hands and feet within 2 days	Quinine	Clopidogrel	Amputated

Hb: Hemoglobin; NA: Not available; M: Male; F: Female

## Case report

A 54 years old male Ugandan presented to Kenyatta National Hospital, a major referral tertiary hospital, in Kenya. He had initially reported with headache and joint pain to a local health facility in Uganda and was diagnosed to have severe *Plasmodium falciparum* malaria infection, and thereafter treatment with intravenous quinine commenced.

Treatment at the facility had been responsive; however two days after the onset, the patient started experiencing episodes of headache, pain, numbness, coldness, and swelling of the extremities. This was followed by darkening of the digits of his toes and hands, which gradually progressed proximally.

On presentation at out institution, he was conscious, alert, oriented, but lethargic and unable to walk. He was a non-smoker and non-alcoholic, and had no history of diabetes, hypertension or any angiopathies. He however had a history of numerous episodes of malaria bouts and a family history of hypertension with his father having succumbed to a stroke.

**Figure 1:**
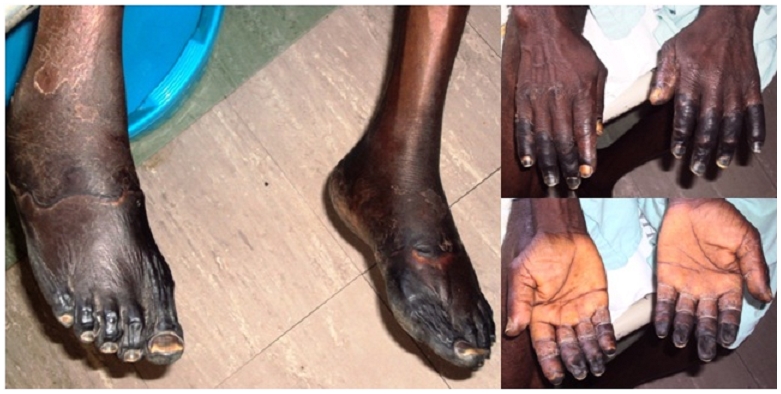
Image showing lower and upper limbs gangrene of the patient prior to surgery

On physical examination, apart from fever, abdominal, cardiovascular, respiratory, and neurological systems were normal. Jaundice, pallor and cyanosis were absent. A right low paramedian scar was noted secondary to appendicectomy conducted in the year 1995. On local examination, the overlying skin cold, dry, wrinkled, and was darkened with a clear line of proximal demarcation (Figure 1). Pain was present in the regions proximal to the discolouration, and absent in the distal regions.

Investigations tested negative for HIV, HBV, HCV, and VDRL on ELISA. Urea, Electrolyte and creatinine tests were normal. Clotting profile showed a prolonged Prothrombin time of 19sec, Prothrombin time index of 74%, and an INR of 1.36. A haemogram showed macrocytic normochromic anaemia (Hb 9.03 g/dL, MCV 89.9fL, MCH 29.5pg, MCHC 27.2g/dL), Hct 33.2%, RBC 3.69x10^12^/L. WBC 4.39x10^9^, Neutrophils 2.11x10^9^ (48.2%), Lymphocytes 1.73x10^9^ (39.5%), Monocytes 0.314x10^9^ (7.16%), Eosinophils 0.193x10^9^ (4.4%), Basophils 0.035x10^9^ (0.796%).

An echocardiogram was normal except for type 1 left ventricular dysfunction and mild tricuspid regurgitation. The Dorsalis pedis pulses showed absence of blood flow, and a diagnosis of Symmetrical peripheral gangrene was made. Management was initiated with clopidogrel, Acetylsalicylic Acid, and Atorvastatin, and quad-amputation recommended. Time was allowed for marginalization of the gangrene, which was noted two months later. Meanwhile fresh frozen plasma was regularly administered to counter suspected thrombophilia.

Following satisfactory marginalization of the gangrene, the patient underwent surgical excision of the necrotic tissue. This involved transmetatarsal amputation of both feet and ray amputation of the hands. The wounds healed well thereafter facilitated by vacuum dressing at -7.5mmHg over one month. He was then referred to plastic surgeons for prosthesis.

## Discussion

Malaria remains a major cause of morbidity and mortality, worldwide [5]. It is an endemic in 42 out of the 46 African countries, and more than 90% of its 1.1 -2.7million worldwide deaths occur within the continent [5].

Of the four causative species, *Plasmodium falciparum* causes 95% of all the cases, and is responsible for most cases of complicated malaria [6]. World Health Organization defines complicated malaria as those accompanied with one or more of the following clinical or laboratory findings i.e. an impaired level of consciousness (Glasgow Coma Scale score 7]. Relevant laboratory findings include severe anemia, hypoglycemia, acidosis, hyperlactatemia, hyperparasitemia (of more than 5%), and renal impairment [7]. This type of malaria has a mortality of more than 10% [8].

Most of the cases reported in table 1, as well as the current case, observed the signs of tissue necrosis within a few days, some as early as two days, following effective anti-malarial therapy. This immediate onset of the ischemia, following initiation of management, and its rapid progression to necrosis, is the striking feature that makes prevention and management of the gangrene difficult. This rapid pattern, furthermore, raises query on the etiology of the gangrene.

It is thought that the cytoadhesive properties of parasite infected erythrocytes that causes the vascular occlusion resulting in cerebral malaria, is the same mechanism of SPG pathogenesis [9].The exact mechanism is however not well understood and only hypotheses have been made as follows.

Heavy parasitemia is thought to cause activation of the compliment system [10], hence triggering the coagulation pathway to cause thrombosis [11]. The parasite is also thought to cause infected erythrocytes to sequestrate within microcirculation by molecular interactions with endothelial receptors mainly intercellular cell adhesion molecule -1 (ICAM-1) [12], endothelial leukocyte adhesion molecule-1 (ELAM-1), vascular cell adhesion molecule –1 (VCAM-1), thrombospondin (TSP) and histidine rich protein (HRP) [13]. This cyto-adherence may then lead to microcirculatory obstruction. Rosetting of uninfected erythrocytes around the infected ones also occurs exacerbating the vascular obstruction caused by the above sequestration [14]. Furthermore, changes in membrane that occur in Plasmodium falciparum infected erythrocytes causes activation of the blood coagulation cascade to cause thrombosis [10,15–17]. During haemolysis, which occurs in malaria infected erythrocytes, negatively charged phosphatidylserines are exposed from the inner leaflet of the erythrocyte membrane bilayer, which trigger the formation of prothrombinase [18]. Furthermore, inflammatory cytokines, especially TNF-a and interleukin-6 [19,20], increase tissue factor expression on mononuclear cells, leading to thrombus formation.

Finally, the life cycle of the Plasmodium has been shown to have an effect on the membrane of infected erythrocyte. As merozoites mature to trophozoite and schizont, the erythrocyte membrane composition changes, leading to appearance of knobs on their surfaces [9]. This alteration of the erythrocyte surface mediates both their adherence to endothelial cells and adhesion to noninfected and other infected erythrocytes. This results in sequestered erythrocytes within the microcirculation, and ultimately vascular obstruction [9].

All these mechanisms may have contributed to the thrombosis, and consequent vascular occlusion, that resulted in the gangrene of our patient. We however question the involvement of the anti-malarial therapy, keeping in mind the acute pattern of onset, following its initiation.

The management of severe malaria is well established [7]. However, the management of specifically patients with malaria and micro-vascular thrombosis is limited [21] to the use of antimalarial chemotherapy, and occasional use of anticoagulants upon symptomatology of ischemia. Quinine is a commonly used antimalarial, especially used in severe malaria [22]. Resistance to the drug is however prevalent within Sub-Saharan Africa, a region which has the highest rates of infection worldwide [23]. Most of the cases on table one, as well as the current case, report the use of quinine, prior to the gangrene, raising question on its involvement in the development and progression of malaria induced gangrene.

Keeping in mind the severity of this complication, its management will therefore require eradication of the parasite with concurrent treatment of the gangrene. The challenge comes in identifying this rare, rapidly progressing complication, before onset of ischemia, as anticoagulants are not usually included in the treatment regime of common malaria. Heparin has commonly been used to counter the thrombosis. The clinical course of *Plasmodium falciparum* malaria is however not positively influenced by its use [19] justifying the use of clopidogrel on our patient. Furthermore, it is contraindicated in patients with severe thrombocytopenia due to the high risk of hemorrhage.

Blood transfusion has also been explored in the management of some of the patients in Table 1. Its benefit, however, remains controversial [24].

The criticality of time cannot be over emphasized considering the extremely short period to onset and rapid progression of tissue necrosis. It has been noted that with timely and correct intervention, the ischemia may resolve before progressing to gangrene. If allowed to progress surgical techniques would be inevitable, the scope of which would depend on the extent and severity of tissue necrosis ranging from debridement to amputation (table 1). Our patient presented with gangrene having already developed, hence missing the opportunity for an early intervention.

## Conclusion

*Plasmodium falciparum* malaria, a very common disease, predisposes a patient to vascular occlusive diseases such as symmetrical peripheral gangrene (SPG). This complication is extremely rare hence almost never anticipated during management of a malaria patient. Furthermore, when it occurs, it progresses extremely rapidly leading to irreversible gangrene, thereby necessitating amputation. Physicians managing malaria patients should therefore be vigilant and on the look-out for SPG in order to intervene promptly and arrest its progression.
